# Antitumor effects of a dual cancer-specific oncolytic adenovirus on colorectal cancer *in vitro* and *in vivo*

**DOI:** 10.3892/etm.2014.2086

**Published:** 2014-11-24

**Authors:** GUOHUA YANG, XIANGWEI MENG, LILI SUN, NINGNING HU, SHUANG JIANG, YUAN SHENG, ZHIFEI CHEN, YE ZHOU, DEXING CHEN, XIAO LI, NINGYI JIN

**Affiliations:** 1 Department of Gastroenterology, The First Hospital of Jilin University, Changchun, Jilin 130021, P.R. China; 2 Institute of Military Veterinary, Academy of Military Medical Sciences, Changchun, Jilin 130122, P.R. China; 3 Jilin Province Qianwei Hospital, Changchun, Jilin 130031, P.R. China; 4 Department of Head and Neck Surgery, Jilin Province Tumor Hospital, Changchun, Jilin 130001, P.R. China; 5 Key Laboratory of Jilin Province for Zoonosis Prevention and Control, Changchun, Jilin 130021, P.R. China

**Keywords:** apoptin, oncolytic adenovirus, SW1116 cell line, GES cell line, colorectal carcinoma

## Abstract

The efficacy and specificity of treatment are major challenges for cancer gene therapy. Oncolytic virotherapy is an attractive drug delivery platform for cancer gene therapy. In the present study, the dual-specific antitumor oncolytic adenovirus, Ad-Apoptin-hTERT-E1a, was used to infect SW1116 human colorectal carcinoma (CRC) cell lines and CT26 mouse-CRC-cell bearing BALB/c mouse models for testing antitumor effects *in vitro* and *in vivo*. The *in vitro* assays revealed that infection with Ad-Apoptin-hTERT-E1a induced a significant cytotoxic effect on the CRC cell line, SW1116; however, the normal human cell line, GES, was only slightly inhibited by the recombinant adenovirus. Acridine orange and ethidium bromide staining and an annexin V assay indicated that infection of SW1116 cells with Ad-Apoptin-hTERT-E1a resulted in a significant induction of apoptosis. Furthermore, western blotting and flow cytometry revealed a decrease in the mitochondrial membrane potential (MMP), the release of cytochrome *c* and the activation of caspase 3, 6 and 7 in Ad-Apoptin-hTERT-E1a-infected SW1116 cells. In the animal models, Ad-Apoptin-hTERT-E1a was shown to significantly inhibit tumor growth and extend the survival times of the animals. Therefore, the experimental results indicated that Ad-Apoptin-hTERT-E1a has potential for application in tumor gene therapy.

## Introduction

Colorectal cancer (CRC) is a malignant tumor that is a major threat to human life due to its high morbidity and mortality rates. The incidence of CRC ranks third among other cancer types, while the mortality rate of CRC ranks fourth worldwide (1). Similar to the majority of cancer types, therapeutic regimens for CRC include traditional therapy, such as aggressive surgery, chemotherapy and radiotherapy. However, surgery itself damages the health of a patient and reduces their resistance to other diseases. Thus, novel therapeutic interventions, particularly biological agents, molecular targeted therapy and gene therapy, have been studied for their ability to treat aspects of CRC that traditional therapy is unable to overcome, including tumor apoptosis-resistance and recurrence (2).

Gene therapy offers a promising strategy for patients who are resistant to traditional therapies, due to its advantage of selectively correcting or eradicating defective tissues and targeting defects in malignant cells (3). The most important issues concerning gene therapy for cancer treatment include the efficiency of transfection and the reliability of expression. Adenovirus vectors have attracted increasing attention in human cancer gene therapy due to their preferential replication in tumor cells (4). An increasing number of clinical trials on oncolytic adenoviruses have been conducted in the last two decades (5). However, non-replicating adenoviruses have little effect in eradicating tumor cells. To overcome such a limitation, the replication competents of adenoviruses that replicate specifically in tumor cells and release virus progeny to further infect and destroy neighboring cancer cells have been developed (6).

Apoptin, a protein derived from chicken anemia virus, has received significant attention as a selective killer of cancer cells (7). Gene expression differences between normal and tumor cells may account for the sensitivity of tumor cells to apoptin. Apoptin is located predominantly in the nucleus of tumor cells, whereas in normal cells, the protein is expressed in the cytoplasm (8). Apoptin expression induces apoptosis in human tumor and transformed cells; however, there has been shown to be little or no cytotoxic effect in a number of normal human cell lines derived from different tissues (9). The combination of apoptin expression with adenovirus-based delivery was selected for cancer therapy due to both molecules having diverse, multiple, yet partly overlapping targets in the cell. Previous studies have indicated that interference with survivin expression facilitates apoptin function. Human telomerase reverse transcriptase (hTERT) is a catalytic subunit of human telomerase that is expressed at a substantially higher level in tumor cells than in normal cells. Telomerase activity influences hTERT expression (10). Thus, hTERT may be a good tumor marker due to the high telomerase activity in ~90% of cancer cells (11). In addition, the hTERT promoter has been used for the tumor-specific expression of transgenes.

In a previous study, an oncolytic adenovirus was combined with the hTERT promoter and the apoptin gene, which functioned as a cancer cell selective apoptosis-inducing gene (12). The oncolytic adenovirus has the ability to inhibit tumor-specific growth and tumor-specific replication (12,13). The present study aimed to determine whether the recombinant Ad-Apoptin-hTERT-E1a vector was able to target CRC cells and induce apoptosis selectively *in vitro* and *in vivo*.

## Materials and methods

### Cell lines, animals and viruses

A human CRC cell line (SW1116), mouse CRC cell line (CT26; syngeneic to C57BL/6 mice) and human gastric epithelium cell line (GES) were obtained from the Type Culture Collection of the Chinese Academy of Sciences (Shanghai, China). The cells were cultured in Dulbecco’s modified Eagle’s medium (Invitrogen Life Technologies, Beijing, China), which was supplemented with 10% heat-inactivated fetal bovine serum (Hyclone Biochemical Product Co. Ltd., Beijing, China), 100 U/ml penicillin and 100 μg/ml streptomycin. The cell lines were passaged for no more than six months following receipt and were subcultured every 48–72 h. A total of 50 female BALB/c mice (age, 6–8 weeks) were purchased from the Experimental Animal Center of the Academy of Military Medical Sciences (Beijing, China) and housed in a pathogen-free facility for all the experiments, following institutional guidelines. The construction and characterization of the dual cancer-specific oncolytic adenovirus, Ad-Apoptin-hTERT-E1a, and the control viruses (Ad-mock, Ad-apoptin and Ad-hTERT-E1a) used in the study have been described previously (12).

### Cell viability assay

Cell viability was determined using a 3-(4,5-dimethylthiazol-2-yl)-2,5-diphenyl tetrazolium bromide (MTT; Sigma-Aldrich, St. Louis, MO, USA) assay, as described previously (13). Briefly, the cells were seeded in a 96-well plate at a density of 5×10^3^/ml, and incubated at 37°C overnight in a humidified environment of 95% air and 5% CO_2_. Cells were infected with disparate concentrations [1, 10 and 100 multiplicity of infection (MOI)] of the recombinant adenoviruses for 12 h. Next, 20 μl MTT (5 mg/ml) was added to each well and incubation was continued at 37°C for 4 h. The culture medium was aspirated and 150 μl dimethylsulfoxide was added to dissolve the insoluble purple formazan product into a colored solution; absorbance was subsequently measured at 490 nm. Thereafter, the absorbance of each well was determined using an automated plate reader (Spectramax 190; Molecular Devices, Sunnyville, CA, USA). The percentage of viable cells was calculated using the background-corrected absorbance as follows: 100 × [(control cells - experimental cells)/control cells]. Cell viability was measured every 12 h over a four-day period. Untreated cells were used as controls.

### Acridine orange and ethidium bromide (AO/EB) staining

Morphological observations of apoptosis were detected by AO/EB staining using a fluorescence microscope (VANOX BX51; Olympus Corporation, Tokyo, Japan). Briefly, the cells were seeded in six-well plates at a density of 1×10^6^ cells per culture well, and cultured for 24 h at 37°C with 5% CO_2_. The cells were infected with the various recombinant adenoviruses (100 MOI) and incubated for 48 h. The treated cells were harvested and washed three times in phosphate-buffered saline (PBS). A 250-μl aliquot was added to a microcentrifuge tube and stained with 4 μl AO-EB (Sigma, St. Louis, MO, USA). Subsequently, 20-μl samples were placed on a microscopic slide and images were collected utilizing the fluorescence microscope. The images were obtained and analyzed using an Image-Pro Plus (version 5.0.2) software program (Media Cybernetics, Inc., Rockville, MD, USA). Untreated cells were used as controls.

### Annexin V/propidium iodide (PI) staining analysis

Annexin V-fluorescein isothiocyanate (FITC)/PI (BD Biosciences, Franklin Lakes, NJ, USA) staining was performed to detect the apoptotic cells by assaying translocated phosphatidylserine (14). In brief, the cells were incubated in six-well plates at a density of 1×10^6^ cells per culture well for 24 h. The cells were cultured for 48 h following infection with the various recombinant adenoviruses (100 MOI). The cells were harvested, washed once with PBS and resuspended in binding buffer. The cells were subsequently stained with FITC-labeled annexin V (Annexin V-FITC Apoptosis Detection kit; BioVision, Inc., Mountain View, CA, USA), according to the manufacturer’s instructions, with simultaneous dye exclusion of PI. The samples were analyzed by flow cytometry (FACScan; BD Biosciences). Untreated cells were used as controls.

### Measurement of the mitochondrial membrane potential (MMP)

The laser dye, rhodamine 123 (Rho123; Sigma-Aldrich), was used to detect the MMP. Briefly, cells (1×10^6^) were cultured for 48 h following infection with the various recombinant adenoviruses (100 MOI). The treated cells were trypsinized and centrifuged at 1,000 × g at 4°C for 5 min. Rho123 (10 μl) was added to the samples, which were subsequently incubated at 37°C for 30 min. Thereafter, the samples were washed with PBS three times. The MMP was quantified by flow cytometry (FACScan), and untreated cells were used as controls.

### Reactive oxygen species (ROS) assay

To quantify the intracellular level of ROS, 2′,7′-dichlorfluorescein-diacetate (DCFH-DA; Sigma) was used. Briefly, cells (1×10^6^) were cultured for 48 h following infection with the various recombinant adenoviruses (100 MOI). The treated cells were trypsinized and centrifuged at 1,000 × g at 4°C for 5 min. ROS levels were determined by treating the cells with 10 μmol/l DCFH-DA at 37°C for 30 min. Data were quantified by flow cytometric analysis (FACScan). Untreated cells were used as controls.

### Caspase analysis

Caspase Activity Assay kits (Beyotime Institute of Biotechnology, Haimen, China) were used to detect the activity levels of caspase-3, 6 and 7 in the treated SW1116 and GES cells. In brief, the cells were infected with the recombinant adenoviruses at 100 MOI for 48 h, trypsinized and washed once with PBS. The cells (1×10^6^) were resuspended in lysis buffer and the total proteins were extracted. The activity levels of caspase-3, 6 and 7 were then analyzed according to the manufacturer’s instructions. The untreated HepG-2 or L02 cells were used as controls.

### Cell fractionation and cytochrome c analysis

Cytoplasmic and mitochondrial fractions were separated, and the cytochrome *c* levels were detected by western blotting. Briefly, the cells (1×10^6^) were infected with the recombinant adenoviruses at 100 MOI for 48 h. The treated cells were then resuspended in lysis buffer [10 mM Tris-HCl (pH 7.8), 1% Nonidet P-40, 10 mM β-mercaptoethanol, 0.5 mM phenylmethylsulfonyl fluoride, 1 mg/ml aprotinin and 1 mg/ml leupeptin] and sheared by passing through a 22-gauge needle. The nuclear fraction was removed by centrifugation at 600 × g for 5 min, and the ‘low-speed’ supernatant was centrifuged at 10,000 × g for 30 min to obtain the mitochondrial fraction (pellet) and the cytosolic fraction (supernatant). The mitochondrial fraction was further lysed in buffer [10 mM Tris (pH 7.4), 150 mM NaCl, 1% Triton X-100 and 5 mM EDTA (pH 8.0)]. The proteins of the extracted samples were separated by SDS-PAGE and transferred onto Hybond-C membranes (GE Healthcare, Pittsburgh, PA, USA). The blots were incubated with rabbit anti-cyto c polyclonal antibody (1:1,000; #4272; Cell Signaling Technology, Inc., Danvers, MA, USA) for 2 h, followed by incubation for 2 h with a horseradish peroxidase labeled goat anti-rabbit IgG secondary antibody (1:1,000; #7074; Cell Signaling Technology, Inc.) labeled with horseradish peroxidase. Signals were visualized using an enhanced chemiluminescence western blotting substrate kit (Pierce Biotechnology, Inc., Rockford, IL, USA).

### Animal experiments

Two methods were used to induce tumors in the mice. In the first model, 1×10^6^ CT26 cells were implanted subcutaneously into the right flank of the BALB/c mice. The tumor-burdened mice were randomly assigned into five groups (five mice per group) following one week of tumor growth. The mice in the first model were treated with the various recombinant adenoviruses, via intratumoral injection at a dose of 1×10^9^ plaque-forming units, in 50 μl saline. The control group received 50 μl saline alone. The injections were administered every two days for the first week (days 6, 8 and 10 following implantation) and once per week for two further weeks (days 17 and 21 following implantation) (15). Tumor size was assessed by caliper measurements of two perpendicular diameters of the implant twice a week. Tumor volume (in cm^3^) was estimated using the following formula: 1/2a × b^2^, where ‘a’ is the long diameter and ‘b’ is the short diameter (in cm). The tumor doubling time (TDT) and the tumor growth delay (TGD) were evaluated at the end of the experiment (16). In the second model, CT26 (1×10^6^) cells were injected into the mice via the tail vein to represent a pulmonary metastasis model. The tumor-burdened mice were randomly assigned into five groups (five mice per group) following one week of tumor growth. The mice were treated intravenously according to the injection protocol of the first model. The animal experiments were conducted in the animal facility of the Institute of Military Veterinary Medicine at the Academy of Military Medical Sciences (Changchun, China), in accordance with governmental and institutional guidelines.

### Statistical analysis

The statistical significance of differences was assessed using one-way analysis of variance, where P<0.05 was considered to indicate a statistically significant difference. Log-rank tests were performed for survival analysis, and data from all the animals were represented in Kaplan-Meier survival plots. All statistical tests were performed using GraphPad Prism 5.0 software (GraphPad, San Diego, CA, USA).

## Results

### Recombinant adenoviruses inhibit the growth of CRC cells

**Figure 1:**
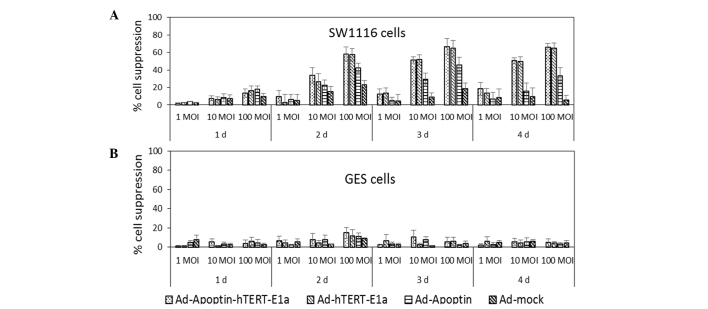
Selective inhibitory effects of Ad-Apoptin-hTERT-E1a on the (A) human CRC cancer SW1116 and (B) GES cell lines. Cells were seeded in 96-well plates (1×10^4^ cells/well) one day prior to infection with various concentrations (1, 10 and 100 MOI) of the indicated adenoviruses. The effects of different MOI and infection times on the viability of the SW1116 and GES cells were assessed. Tumor viability was measured every day over a four-day period using the 3-(4,5-dimethylthiazol-2-yl)-2,5-diphenyl tetrazolium bromide colorimetric assay, and all measurements were performed in triplicate. Data are presented as the mean ± standard deviation. In the SW1116 human CRC cells, Ad-Apoptin-hTERT-E1a infection resulted in significant growth inhibition. By contrast, in the GES cells, the recombinant adenoviruses did not inhibit cell growth. MOI, multiplicity of infection; hTERT, human telomerase reverse transcriptase.

An MTT assay was used to measure the viability of the cells infected with the various adenoviruses (17). As shown in Fig. 1A, in the early stages of infection, the adenovirus did not cause significant inhibition of the CRC cells. However, as the infection time extended, Ad-Apoptin-hTERT-E1a, Ad-hTERT-E1a and Ad-Apoptin were shown to inhibit SW1116 tumor cells, with the level of inhibition increasing with an increased infection dose. In the SW116 cells infected with Ad-mock, after two days, the infective doses of 1, 10 and 100 MOI induced cell growth inhibition of 0–5, 15–20 and 20–25%, respectively. The Ad-mock treated group demonstrated a gradual attenuation of inhibition. Although cells infected with Ad-Apoptin induced a higher level of inhibition compared with the Ad-mock-treated group, the level of inhibition decreased after three days due to the absence of the replication gene. By contrast, cells treated with the replication-competent adenoviruses (Ad-Apoptin-hTERT-E1a and Ad-hTERT-E1a) showed significant suppression of cell growth, which correlated with the infection doses. When infected with 1, 10 and 100 MOI Ad-Apoptin-hTERT-E1a or Ad-hTERT-E1a, after two days, the cell growth was inhibited by 5–10, 30–35 and 55–60%, respectively. Inhibition in the Ad-Apoptin-hTERT-E1a and Ad-hTERT-E1a groups peaked at a MOI of 100, with the suppression rates between 70 and 75% after three days. The inhibition induced by Ad-Apoptin-hTERT-E1a was slightly higher compared with that of Ad-hTERT-E1a. In addition, the cell growth suppression observed following infection with Ad-Apoptin-hTERT-E1a or Ad-hTERT-E1a at 10 MOI was similar to that following infection with 100 MOI Ad-Apoptin. Inhibition by the combined replication-competent adenoviruses decreased a little after four days. However, in the normal GES cells (Fig. 1B), regardless of the recombinant adenovirus infection time and infection dose, cells treated with the recombinant adenoviruses did not show a marked inhibitory effect. Thus, the adenoviruses with the dual cancer-specific genes were more effective compared with the normal replication-incompetent adenoviruses in inhibiting cancer cell growth. The interaction of infection time and MOI was complex and synergistic, and cell viability revealed a non-rigorous dependent association with the two factors. Therefore, the *in vitro* experiments were performed 48 h following infection at a MOI of 100.

### Recombinant adenoviruses induce the apoptosis of CRC cells

**Figure 2:**
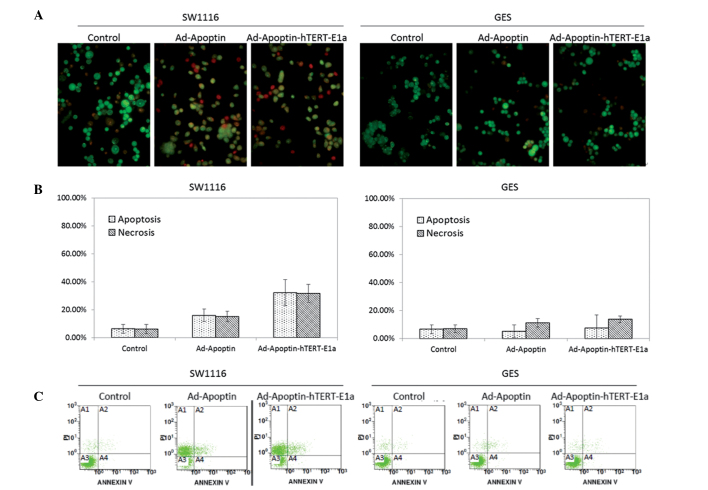
Selective induction of apoptosis in colorectal cancer cells by Ad-Apoptin-hTERT-E1a. (A) Fluorescence images (magnification, ×100) show the morphological changes of the recombinant adenovirus-infected SW1116 and GES cells stained with acridine orange and ethidium bromide. (B) Image-Pro Plus analysis of the SW1116 and GES cells infected with the recombinant adenoviruses was performed to quantify the apoptotic and necrotic cell populations. Microscopic images were captured and analyzed by the Image-Pro Plus software program. Data are expressed as the mean ± standard deviation. (C) Flow cytometric analysis of the SW1116 and GES cells infected with the recombinant adenoviruses. hTERT, human telomerase reverse transcriptase; PI, propidium iodide.

AO dye is unable to permeate the intact cell membrane, which stains live cells with bright green fluorescence, while EB can only enter the membrane of damaged cells and stains the nuclei orange (18). Images of AO/EB staining and the results of Image-Pro Plus analysis are shown in Fig. 2A and B. Fig. 2A compares the morphological changes for SW1116 and GES cells treated with the recombinant adenoviruses. Live cells are shown with a normal green nucleus; early apoptotic cells have a bright green nucleus with condensed or fragmented chromatin; late apoptotic cells exhibit condensed and fragmented orange chromatin; and cells that have died from direct necrosis have structurally normal orange nuclei. As shown in Fig. 2B, the proportion of necrotic and apoptotic cell populations in the control and treated SW1116 or GES cells was significantly different. In the SW1116 cells, infection with Ad-Apoptin-hTERT-E1a resulted in apoptosis (32.2%) and necrosis (31.5%). However, the proportion of cells undergoing apoptosis or necrosis in the Ad-Apoptin-hTERT-E1a-treated GES cells was similar to the uninfected control GES cells. The percentage of apoptotic cells following recombinant adenovirus treatment was quantified by flow cytometry. As shown in Fig. 2C, in contrast to the GES cells, infection with Ad-Apoptin-hTERT-E1a resulted in the apoptosis of SW1116 cells. These results indicated that Ad-Apoptin-hTERT-E1a specifically induced apoptosis in CRC cells.

### Recombinant adenoviruses induce apoptosis via the mitochondrial pathway

**Figure 3:**
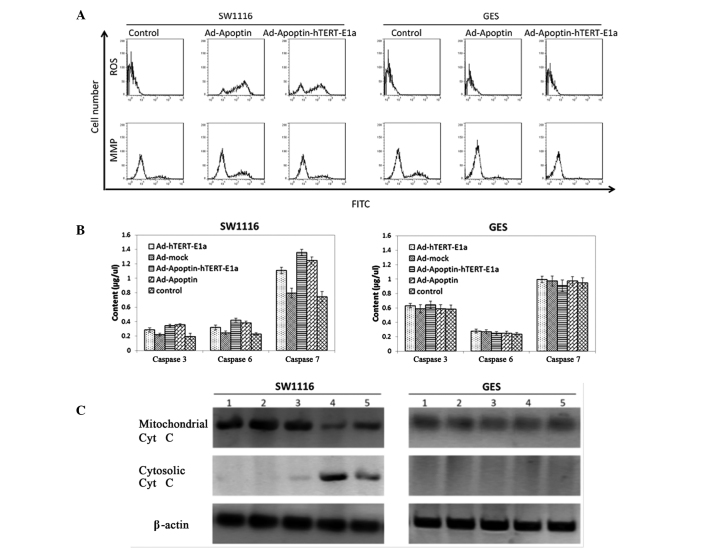
Analysis of the mitochondrial permeability transition of the recombinant adenovirus-treated SW1116 cells and GES cells. (A) Flow cytometric determination of the MMP and level of ROS in the SW1116 and GES cells infected with the recombinant adenoviruses. (B) Detection of caspase activity in the recombinant adenovirus-treated SW1116 and GES cells. (C) Expression of cytochrome *c* in the recombinant adenovirus-treated SW1116 and GES cells was detected by western blotting. FITC, fluorescein isothiocyanate; hTERT, human telomerase reverse transcriptase; ROS, reactive oxygen species; MMP, mitochondrial membrane potential.

The effects of the recombinant adenoviruses on the MMP and ROS levels in SW1116 cells were determined. As shown in Fig. 3A, Ad-Apoptin-hTERT-E1a-infected SW1116 cells showed a significant increase in ROS levels compared with the untreated control group. In the GES cells, infection with Ad-Apoptin-hTERT-E1a resulted in a slight increase in the level of ROS compared with the control group. The results of MMP analysis were similar to that of the ROS detection. SW1116 cells infected with Ad-Apoptin-hTERT-E1a showed a significant decrease in the MMP, while in GES cells, Ad-Apoptin-hTERT-E1a treatment did not result in a MMP decrease, as compared with the control group (Fig. 3A). In addition, the activity levels of caspases were determined (Fig. 3B). Infection of SW1116 cells with Ad-Apoptin-hTERT-E1a caused a marked increase in the activity levels of caspses-3, 6 and 7. By contrast, caspase activity was not detected in the GES cells infected with the recombinant adenoviruses. Furthermore, significant quantities of cytochrome *c* were detected in the cytosol of the Ad-Apoptin-hTERT-E1a-infected SW1116 cells (Fig. 3C). The levels of cytochrome *c* in the cells treated with Ad-Apoptin-hTERT-E1a were more evident than in the control groups. However, Ad-Apoptin-hTERT-E1a exhibited no significant effects on cytochrome *c* release in the GES cells (Fig. 3C). These results indicated that the specific apoptosis of SW1116 cells triggered by Ad-Apoptin-hTERT-E1a was associated with the release of mitochondrial cytochrome *c*, a decrease in the MMP and an increase in the levels of ROS.

### Ad-Apoptin-hTERT-E1a suppresses subcutaneous primary tumor growth

**Figure 4:**
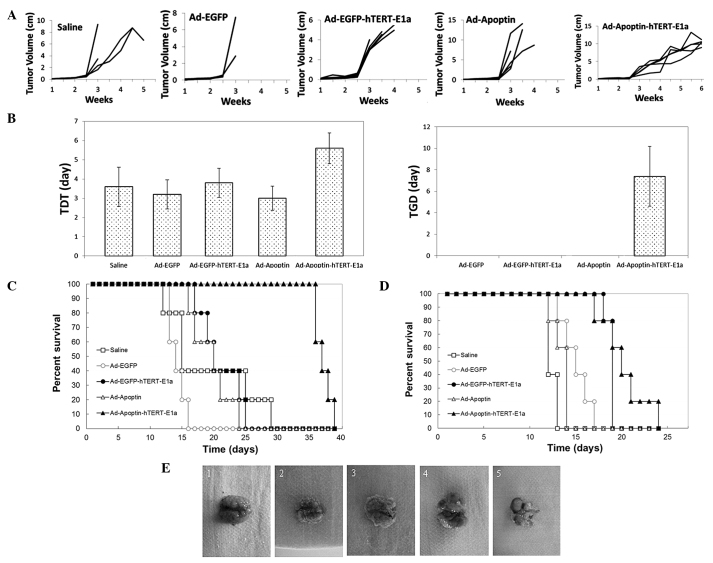
Ad-Apoptin-hTERT-E1a suppression of colorectal cancer in the BALB/c mice model. (A) Tumor growth kinetics of the mice that received recombinant adenoviruses by intratumoral injections. (B) TDT and TGD analyses of the mice that received intratumoral injections of the recombinant adenoviruses. Survival curves of the mice treated (C) intratumorally and (D) intravenously. (E) Representative images of the lungs from the control and treatment groups of the mice treated intravenously. The day of the first injection was considered as day 0. 1, Control; 2, Ad-EGFP; 3, Ad-EGFP-hTERT-E1a; 4, Ad-Apoptin; 5, Ad-Apoptin-hTERT-E1a; hTERT, human telomerase reverse transcriptase; EGFP, enhanced green fluorescent protein; TDT, tumor doubling time; TGD, tumor growth delay.

The antitumor potential of Ad-Apoptin-hTERT-E1a was examined in a mouse CT26 tumor model. The growth kinetics of the tumors following treatment are shown in Fig. 4A. Compared with the saline control and the Ad-enhanced green fluorescent protein (EGFP) group, the growth of the tumors in the recombinant adenovirus groups was suppressed. However, following three injections, the tumors infected with Ad-EGFP, Ad-EGFP-hTERT-E1a and Ad-Apoptin resumed their usual growth. By contrast, the majority of Ad-Apoptin-hTERT-E1a-infected tumors grew slowly. Furthermore, the intratumoral injection of Ad-Apoptin-hTERT-E1a significantly increased the TDT and TGD. When compared with the saline control groups, Ad-Apoptin-hTERT-E1a significantly increased the TDT from 3.6 to 5.6 days (Fig. 4B; P<0.05). In addition, treatment with Ad-Apoptin-hTERT-E1a delayed tumor growth by 7.4 days, whereas the other recombinant adenoviruses exhibited no tumor delaying effects (Fig. 4B; P<0.05). The ability of the recombinant adenoviruses to prolong the mean survival times of the tumor-bearing mice was also investigated. As shown in Fig. 4C, the mice treated with Ad-Apoptin-hTERT-E1a had the longest survival times. The mean survival times were 19.2±3.3 days for the saline-treated mice, 14.2±0.6 days for the Ad-EGFP-infected mice, 21±1.5 days for the Ad-EGFP-hTERT-E1a-infected mice, 19.6±1.4 days for the Ad-Apoptin-infected mice and 37.2±0.6 days for the Ad-Apoptin-hTERT-E1a-infected mice. The results indicated that intratumoral injections of Ad-Apoptin-hTERT-E1a conferred significant survival benefits *in vivo*.

### Systemic delivery of Ad-Apoptin-hTERT-E1a reduces the number of metastatic lung nodules

Survival analysis revealed that Ad-Apoptin-hTERT-E1a treatment significantly increased the survival times of the mice in the lung metastasis model group when compared with treatment using the other recombinant adenoviruses or with saline (Fig. 4D). The mean survival times were 12.4±0.2 days for the saline-treated mice, 15±0.7 days for the Ad-EGFP-infected mice, 18.8±0.2 days for the Ad-EGFP-hTERT-E1a-infected mice, 13.4±0.4 days for the Ad-Apoptin-infected mice and 20.2±1.2 days for the Ad-Apoptin-hTERT-E1a-infected mice. As shown by the representative metastatic nodules in Fig. 4E, Ad-Apoptin-hTERT-E1a significantly decreased the tumor burden of the mice. The lungs of the mice infected with Ad-Apoptin-hTERT-E1a exhibited minimal metastatic nodules, whereas the lungs from the control or treated groups exhibited severe metastasis. Therefore, systemic delivery of Ad-Apoptin-hTERT-E1a was shown to significantly reduce the tumor burden and provide survival benefits in a lung metastatic cancer model.

## Discussion

Cancer is a serious threat to public health. The pathogenesis of cancer is that normal cells are transformed into a malignant cells, in which apoptosis is reduced (19). Thus, promoting the apoptosis of cancer cells plays a vital role in oncotherapy. Oncolytic adenoviruses are promising tools in cancer therapeutics due to their ease of manipulation and multiple, distinct anticancer mechanisms, including direct lysis, apoptosis induction, expression of toxic proteins, autophagy and the inhibition of protein synthesis, as well as the induction of antitumoral immunity (20,21). Assessing therapeutic genes to insert into the viral genome has been a major focus in cancer virotherapy, and the types of transgenes considered for this purpose have included tumor suppressor, proapoptotic, antiangiogenic, ‘suicide’ and immunomodulatory genes (13). Apoptin is the VP3 protein of chicken infectious anemia virus. The protein is p53-independent, Bcl-2-insensitive and apoptotic (22). Apoptin resides in the cytoplasm of normal cells, whereas it localizes in the nucleus of cancer cells (23). The nuclear translocation of apoptin largely depends on phosphorylation (14). Apoptin has the specific ability to kill human cancer cells and transform cells without interfering with normal cell proliferation (15). Apoptin induces various types of human cancer cell lines to undergo apoptosis via classical apoptotic pathways (16).

In cancer gene therapy, the specificity of the promoter that controls the expression of an exogenous gene to target cells determines the treatment validity (17). Telomeres are repeated DNA sequences that provide protection for chromosomes (18). Telomerase, in which hTERT functions as the catalytic protein, adds telomere repeats to chromosomes (19). Telomerase activity is closely associated with hTERT expression (24). The activity of the hTERT promoter has been associated with cancer and has been detected in a number of invasive cancer types; however, the promoter is repressed in normal somatic tissues or benign tumors (25). The hTERT promoter has been used to drive the expression of a number of genes for cancer therapeutics (26,27). In a previous study, a tumor-specific apoptosis-inducing gene (apoptin) and a cancer-specific promoter (hTERTp) were inserted into the RAPAd adenoviral vector to obtain a novel, dual-specific antitumor oncolytic adenovirus, Ad-Apoptin-hTERT-E1a, as well as the control recombinant adenoviruses, Ad-Apoptin, Ad-EGFP and Ad-EGFP-hTERT-E1a (13). In the present study, the antitumor effects of these novel oncolytic viruses were evaluated in CRC cells *in vitro* and *in vivo*. In order to avoid the influence of EGFP gene expressed by Ad-EGFP on the fluorescence experiment, the Ad-EGFP virus was not used in the *in vitro* studies. While, to facilitate the *in vivo* imaging studies (data not shown), Ad-EGFP was used in the animal experiments.

As shown in Fig. 1, the cell viability showed a non-rigorous dependence on the infection time and MOI. With increasing infection times and increasing infective doses, the inhibitory effects on SW1116 cells treated with Ad-Apoptin-hTERT-E1a became more evident than in cells infected with the other recombinant adenoviruses. By contrast, Ad-Apoptin-hTERT-E1a had a limited inhibitory effect on GES cells. The AO/EB staining assay was used to analyze cell death and quantify the relative proportions of live, apoptotic and necrotic recombinant adenovirus-infected cells (Fig. 2A and B). The results indicated that Ad-Apoptin-hTERT-E1a significantly induced apoptosis and necrosis in SW116 cells, but had no effect on the normal GES cells. In addition, annexin V assays indicated that Ad-Apoptin-hTERT-E1a was able to suppress the growth of SW1116 cells through the induction of apoptosis (Fig. 2C), while the normal GES cells showed little sensitivity to the recombinant adenovirus. Furthermore, Ad-Apoptin-hTERT-E1a caused an apparent increase in the levels of ROS, significantly reduced the MMP and activated caspases in the SW1116 cells (Fig. 3A and B). The release of cytochrome *c* was also detected in Ad-Apoptin-hTERT-E1a-infected SW1116 cells (Fig. 3C). By contrast, the effects of Ad-Apoptin-hTERT-E1a on GES cells were minimal. Therefore, the results confirmed the previous observations that Ad-Apoptin-hTERT-E1a has the potential to specifically kill CRC cells by inducing the apoptosis pathway.

The *in vivo* antitumor activities of the recombinant adenoviruses were also evaluated in a CRC mouse model, which further confirmed the efficacies observed *in vitro*. As shown in Fig. 4A–C, Ad-Apoptin-hTERT-E1a exhibited significant antitumor effects compared with the other recombinant adenoviruses in the primary tumor model. Injection of Ad-Apoptin-hTERT-E1a directly into the tumors resulted in a complete response to treatment and the longest mean survival time, which were the best outcomes compared with the results from the other recombinant adenovirus-treated groups. In the *in vivo* experiments, the antitumoral effects of Ad-Apoptin-hTERT-E1a were also observed on metastatic tumors. The data indicated that Ad-Apoptin-hTERT-E1a inhibited the formation of metastatic tumors successfully (Fig. 4D and E). Furthermore, no toxic effects were observed following the injection of Ad-Apoptin-hTERT-E1a.

In conclusion, the effects of Ad-Apoptin-hTERT-E1a on the CRC SW1116 cell line were investigated *in vitro*. The results demonstrated that Ad-Apoptin-hTERT-E1a specifically replicated in human SW1116 tumor cells and restricted the growth of these cells selectively, while showing no adverse effects on GES cells. In addition, the results obtained from the *in vivo* tumor model indicated that Ad-Apoptin-hTERT-E1a not only inhibited primary transplanted tumors, but also played a key role in suppressing the metastasis of tumors. These results highlight the need for further evaluation of Ad-Apoptin-hTERT-E1a as a novel class of drugs for the clinical treatment of CRC.
